# Beyond screening: Sensory impairment and arthritis as a mandate for integrated geriatric care

**DOI:** 10.1111/ggi.70179

**Published:** 2025-09-18

**Authors:** Yi‐Ching Chu, Chao‐Chun Huang

**Affiliations:** ^1^ Department of Ophthalmology Far Eastern Memorial Hospital New Taipei City Taiwan; ^2^ Department of Physical Medicine and Rehabilitation Far Eastern Memorial Hospital New Taipei City Taiwan; ^3^ Department of Physical Therapy and Assistive Technology National Yang Ming Chiao Tung University Taipei Taiwan

Dear Editor,

We commend Wang *et al*. for their robust, population‐based study in *Geriatrics & Gerontology International*, which provides compelling longitudinal evidence for the independent and joint associations of vision and hearing impairments with arthritis, highlighting sensory health as an underrecognized determinant of musculoskeletal well‐being in older adults.^1^ Specifically, their report that dual sensory impairment (DSI) nearly doubled the odds of prevalent arthritis (odds ratio 2.03) and increased incident risk by 25% (hazard ratio 1.25) is a crucial contribution.^1^ The dose–response relationship they observed aligns with the established understanding of DSI as a formidable geriatric syndrome that synergistically amplifies the risk for devastating outcomes, including dementia and accelerated functional decline.^2^, ^3^, ^4^ Collectively, these findings demand an urgent shift from simple screening toward actionable, integrated intervention strategies that bridge the gap between diagnosis and comprehensive management.

While we strongly support the authors' call for routine sensory screening, the critical question is what happens *after* a diagnosis is made. The co‐occurrence of sensory loss and arthritis creates a vicious cycle of immobility. Arthritis‐related pain is a well‐documented barrier to physical activity, a cornerstone of non‐pharmacological management as recommended by 2018 European Alliance of Associations for Rheumatology (EULAR) guidelines.^5^ Sensory impairments erect additional, formidable barriers: visual impairment increases the fear of falling, while hearing impairment hinders communication during group exercise.^4^ Consequently, patients with DSI and arthritis are less able to engage in the very intervention most critical for their joint disease. This feedback loop accelerates deconditioning and functional decline, ultimately leading to poorer quality of life and increased healthcare utilization—an urgent issue for aging societies across Asia.

This clinical challenge highlights a critical flaw in our healthcare systems: persistent fragmentation despite increasing multimorbidity in aging populations. As a recent qualitative study from Beijing reveals, patients with multiple conditions often struggle to navigate a tiered system of disconnected specialists, leading to uncoordinated and inefficient care.^6^ This fragmented, disease‐oriented model fails to address the complex functional interplay between conditions. The findings of Wang *et al*., therefore, serve as a powerful, data‐driven indictment of this outdated paradigm.^1^

The only way to break this vicious cycle is to move beyond fragmented services toward integrated, person‐centered geriatric care. Such a model, guided by frameworks like the Chronic Care Model, shifts the focus from treating discrete diseases to achieving the patient's functional goals.^7^ A scoping review of care models for multimorbidity confirms the need for a coordinated, multidisciplinary team that can develop a single, shared care plan.^8^ This plan would not treat arthritis and DSI in isolation but would explicitly address their interactions. For instance, a physiotherapist could develop low‐vision‐adapted exercise regimens, while an audiologist could optimize hearing aids for group rehabilitation. An occupational therapist would further support this by conducting a home safety assessment that accounts for both mobility and sensory deficits.

In conclusion, the profound contribution of Wang *et al*. is in providing population‐level evidence that underscores the urgent need to redesign care for older adults with complex, interacting morbidities. As healthcare systems across Asia face unprecedented demographic shifts, the necessity for integrated models is not just a theoretical ideal but a practical imperative.^9^ As one of the leading journals in geriatric care, *Geriatrics & Gerontology International* provides an ideal platform to catalyze the research, discussion, and implementation of such models. By fostering this cross‐disciplinary collaboration, we can move beyond isolated disease management toward evidence‐based, patient‐centered strategies that preserve function, independence, and quality of life.

## Author contributions

Y‐CC conceived the study and drafted the manuscript. C‐CH provided critical revision and supervised the project. Both authors have read and approved the final manuscript.

## Funding

The authors received no specific funding for this work.

## Disclosure statement

The authors declare that they have no conflicts of interest.

## Ethics statement

This article is a commentary on a previously published study and does not involve human participants or animals. Therefore, institutional review board approval was not required.

## Patient consent statement

Not applicable, as this study did not involve any patients.

## Clinical trial registration

Not applicable.

## Data Availability

Data sharing is not applicable to this article as no new data were created or analyzed in this study.

## References

[1] Wang X , Si H , Li Y *et al*. Independent and joint associations of vision and hearing impairments with arthritis: findings from a population‐based study. Geriatr Gerontol Int 2025; 25: 1370–1378. 10.1111/ggi.70156. 40878037

[2] Wang Q , Zhang S , Wang Y , Zhao D , Chen X , Zhou C . The effect of dual sensory impairment and multimorbidity patterns on functional impairment: a longitudinal cohort of middle‐aged and older adults in China. Front Aging Neurosci 2022; 14: 807383. 35462686 10.3389/fnagi.2022.807383 PMC9028763

[3] Hwang PH , Longstreth WT , Brenowitz WD *et al*. Dual sensory impairment in older adults and risk of dementia from the GEM study. Alzheimers Dement (Amst) 2020; 12: e12054. 32671180 10.1002/dad2.12054 PMC7340796

[4] Kong H , Shin K , Won CW . Association of Dual Sensory Impairment with declining physical function in community‐dwelling older adults. Int J Environ Res Public Health 2023; 20: 3989. 36834243 10.3390/ijerph20043546 PMC9964928

[5] Rausch Osthoff AK , Niedermann K , Braun J *et al*. 2018 EULAR recommendations for physical activity in people with inflammatory arthritis and osteoarthritis. Ann Rheum Dis 2018; 77: 1251–1260. 29997112 10.1136/annrheumdis-2018-213585

[6] You C , Zhao J , Fan T *et al*. Navigating fragmented care: a qualitative study on multimorbidity management challenges in Beijing's tiered healthcare system. BMC Prim Care 2025; 26: 15. 40877757 10.1186/s12875-025-02967-y PMC12392530

[7] Wagner EH . Chronic disease management: what will it take to improve care for chronic illness? Eff Clin Pract 1998; 1: 2–4. 10345255

[8] Struckmann V , Leijten FRM *et al*. Relevant models and elements of integrated care for multi‐morbidity: results of a scoping review. Health Policy 2018; 122: 28–39. 10.1016/j.healthpol.2017.08.008 29031933

[9] Tham TY , Tran TL *et al*. Integrated health care systems in Asia: an urgent necessity. Clin Interv Aging 2018; 13: 2427–2429. 10.2147/CIA.S185048 PMC6298881 30587945

